# Honokiol suppresses lung tumorigenesis by targeting EGFR and its downstream effectors

**DOI:** 10.18632/oncotarget.10759

**Published:** 2016-07-21

**Authors:** Jung Min Song, Arunkumar Anandharaj, Pramod Upadhyaya, Ameya R. Kirtane, Jong-Hyuk Kim, Kwon Ho Hong, Jayanth Panyam, Fekadu Kassie

**Affiliations:** ^1^ Masonic Cancer Center, University of Minnesota, Minneapolis, MN 55455, USA; ^2^ Department of Pharmaceutics, University of Minnesota, Minneapolis, MN 55455, USA; ^3^ Institute for Therapeutics Discovery and Development, University of Minnesota, Minneapolis, MN 55414, USA; ^4^ Department of Veterinary Clinical Sciences, College of Veterinary Medicine, University of Minnesota, Saint Paul, MN 55108, USA

**Keywords:** chemoprevention, honokiol, 4-(methylnitrosamino)-1-(3-pyridyl)-1-butanone, lung tumor, EGFR

## Abstract

Since epidermal growth factor receptor (EGFR) is commonly deregulated in pre-malignant lung epithelium, targeting EGFR may arrest the development of lung cancer. Here, we showed that honokiol (2.5–7.5 μM), a bioactive compound of *Magnolia officinalis*, differentially suppressed proliferation (up to 93%) and induced apoptosis (up to 61%) of EGFR overexpressing tumorigenic bronchial cells and these effects were paralleled by downregulation of phospho-EGFR, phospho-Akt, phospho-STAT3 and cell cycle-related proteins as early as 6–12 h post-treatment. Autocrine secretion of EGF sensitized 1170 cells to the effects of honokiol. Molecular docking studies indicated that honokiol binds to the tyrosine kinase domain of EGFR although it was less efficient than erlotinib. However, the anti-proliferative and pro-apoptotic activities of honokiol were stronger than those of erlotinib. Upon combinatory treatment, honokiol sensitized bronchial cells and erlotinib resistant H1650 and H1975 cells to erlotinib. Furthermore, in a mouse lung tumor bioassay, intranasal instillation of liposomal honokiol (5 mg/kg) for 14 weeks reduced the size and multiplicity (49%) of lung tumors and the level of total- and phospho-EGFR, phospho-Akt and phospho-STAT3. Overall, our results indicate that honokiol is a promising candidate to suppress the development and even progression of lung tumors driven by EGFR deregulation.

## INTRODUCTION

Lung cancer is the leading cause of cancer-related death in the USA. The American Cancer Society estimated that 224,390 new cases of lung cancer would be diagnosed in the United States by the end of 2016, and there would be 158,090 lung cancer-related deaths, which would account for approximately 27% of all cancer deaths [1]. Therefore, novel and effective preventive and therapeutic agents should be developed to combat this major health problem. In particular, drugs that selectively target molecular pathways differentially activated/overexpressed in cancer cells and regulate the growth and progression of lung cancer would be attractive.

Honokiol is a biologically active phenolic compound isolated from the root and bark of *Magnolia officinalis*. This plant has been used in traditional Chinese medicine for thousands of years for the treatment of various ailments because of its muscle relaxant, anti-oxidative, anti-inflammatory, anti-allergic and anti-bacterial activities, reflecting a long and safe record of usage [2, 3]. Recently, honokiol has emerged as a promising anticancer agent [4–8].

Deregulation of EGFR, a member of the erbB family of tyrosine kinase receptor proteins, is the most common genetic change driving the development of non-small cell lung cancer (NSCLC) [9, 10]. It is overexpressed in up to 62% of NSCLC and mutated in about 40% of adenocarcinomas and 30% of adenosquamous NSCLC [9, 10]. EGFR family members are deregulated in cancer cells by three fundamental mechanisms: activating gene mutations, increased gene copy number, and altered ligand expression with possible formation of autocrine loops [11, 12]. Receptor-ligand interaction results in the formation of homodimers or heterodimers, activation of the intrinsic kinase domain, and phosphorylation of specific tyrosine residues which leads to the activation of downstream signaling pathways including PI3K/Akt, STAT3, and RAS/RAF/MEK pathways [11]. Frequent overexpression and activation of EGFR has also been reported in histologically normal and hyperplastic bronchial epithelium from smokers [13], bronchial preneoplasia [14], and in histologically normal bronchial epithelium adjacent to lung adenocarcinomas [15], indicating the possibility that alterations in EGFR signaling represent an early event and may represent a target for chemopreventive agents.

Here, we report the lung cancer chemopreventive activities of honokiol and the mechanisms involved. Honokiol induced differential anti-proliferative and apoptotic activities in tumorigenic bronchial cells overexpressing EGFR, and its efficacy was stronger than that of the EGFR tyrosine kinase inhibitor erlotinib. Honokiol also significantly reduced the number of lung tumors induced by 4-(methylnitrosamino)-1-(3-pyridyl)-1-butanone (NNK) in A/J mice and these effects were paralleled by decreased phosphorylation of EGFR and related proteins.

## RESULTS

### Honokiol differentially reduced the growth of tumorigenic (1170) bronchial cells

**Figure 1:**
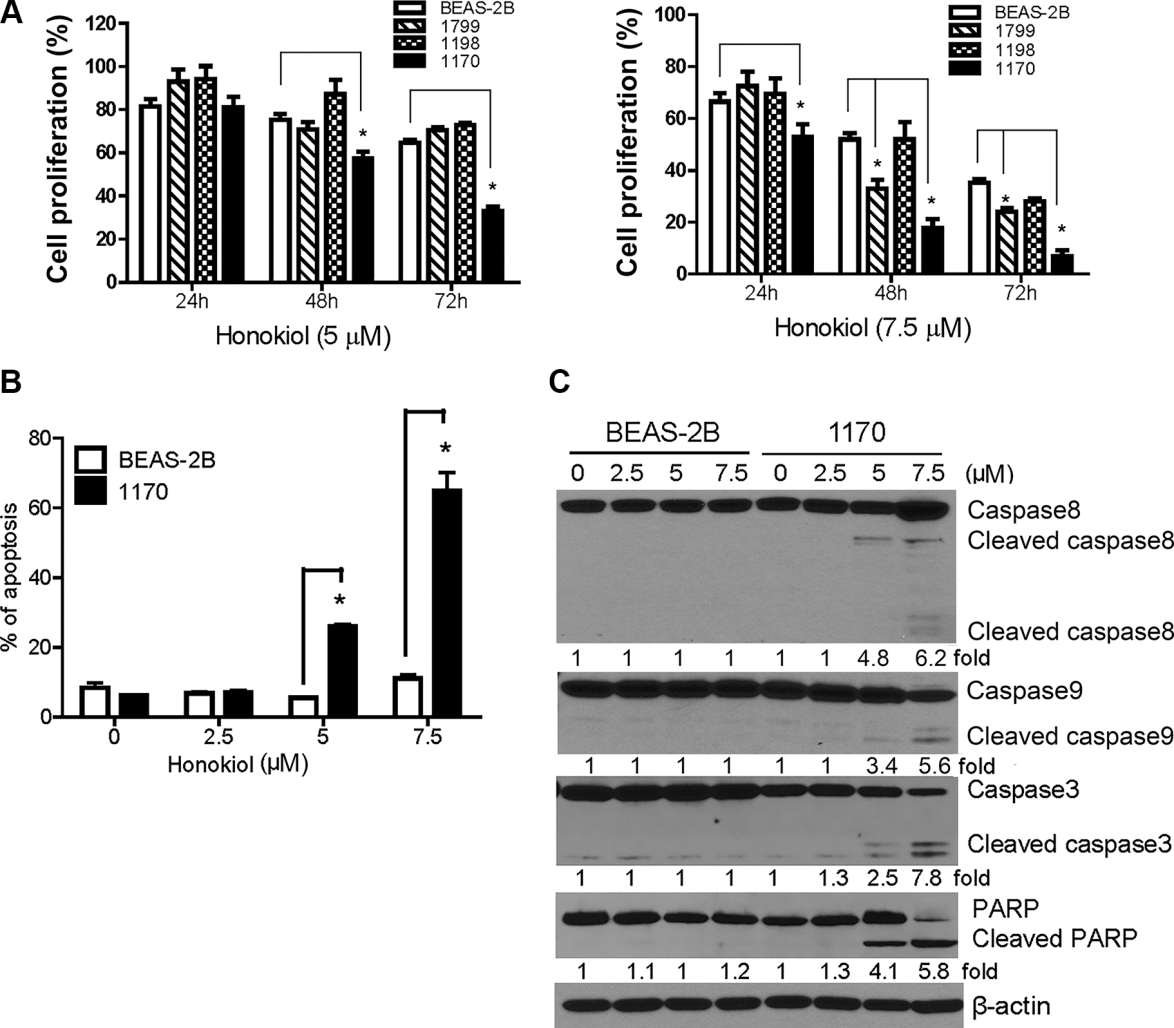
Honokiol induced differential anti-proliferative and pro-apoptotic effects in tumorigenic bronchial (1170) cells (**A**) Dose- and time-dependent anti-proliferative effects of honokiol in bronchial cells. MTT assays were performed in DMSO- or honokiol-treated parental BEAS-2B cell line and its premalignant (1799 and 1198) and tumorigenic (1170) derivatives as described in Materials and Methods and the data were presented as percentage mean ± SD of cell viability compared to untreated cells. Data were obtained from three independent experiments. (**B**) Bar graph showing the mean percentage of apoptotic BEAS-2B and 1170 cells obtained from three independent measurements. Cells were treated with different concentrations of honokiol (0, 2.5, 5, or 7.5 μM) for 72 h, stained with Annexin V and PI, and the percentage of apoptotic cells determined by flow cytometry. *P < 0.05 compared to untreated control. (**C**) Representative Western immunoblotting results showing differential cleavage of caspase-8, caspase-9, caspase-3, and PARP in 1170 cells treated with honokiol (0, 2.5, 5 and 7.5 μM) for 72 h. Three independent immunoblot assays were performed from different samples as described in the Materials and Methods section.

Human bronchial cells at different stages of transformation- immortalized BEAS-2B, premalignant 1179 and 1198 and tumorigenic 1170 bronchial cells- were treated with honokiol and cell proliferation was determined by MTT assay. As shown in Figure 1A, treatment of 1170 bronchial cells with honokiol at concentrations of 5 and 7.5 μM significantly reduced cell proliferation by 19% and 43%, 47% and 67%, and 82% and 93% at 24, 48 and 72 h, respectively. Under identical conditions, the lower concentration of the drug did not cause significant growth inhibitory effects in the parental BEAS-2B cell line or its premalignant derivatives, whereas the higher concentration of honokiol significantly reduced the viability of 1799 cells though the effects were not as strong as those observed in 1170 cells. MTT assays showed that liposomal honokiol has similar anti-proliferative effects as that of the non-liposomal (free) form of the drug (Data not shown).

To examine the differential pro-apoptotic activities of honokiol towards the tumorigenic cell line, BEAS-2B and 1170 cells were treated with the drug as described above, stained with annexin V and propidium iodide and analyzed by flow cytometry. The results were confirmed by Western immunoblotting-based detection of apoptosis-related proteins. In line with the MTT assay results, exposure of 1170 cells to 5 and 7.5 μM of honokiol dramatically increased the percentage of apoptotic cells (early and late apoptotic cells) by about 26% and 61%, respectively, compared to the 5% and 12% increase in BEAS-2B cells (Figure 1B). Western immunoblotting assay revealed the cleavage of full length caspase-3, −8 and −9 as well as PARP, which are instrumental in triggering apoptosis, in 1170 cells treated with 5 and 7.5 μM of honokiol, whereas no such effects were observed in BEAS-2B cells (Figure 1C). Overall, these results showed that honokiol differentially reduced the survival of tumorigenic 1170 cells while it only induced minimal effects in parental normal cells.

### Honokiol inhibited the EGFR signaling pathway in 1170 cells in a dose- and time-dependent manner

**Figure 2:**
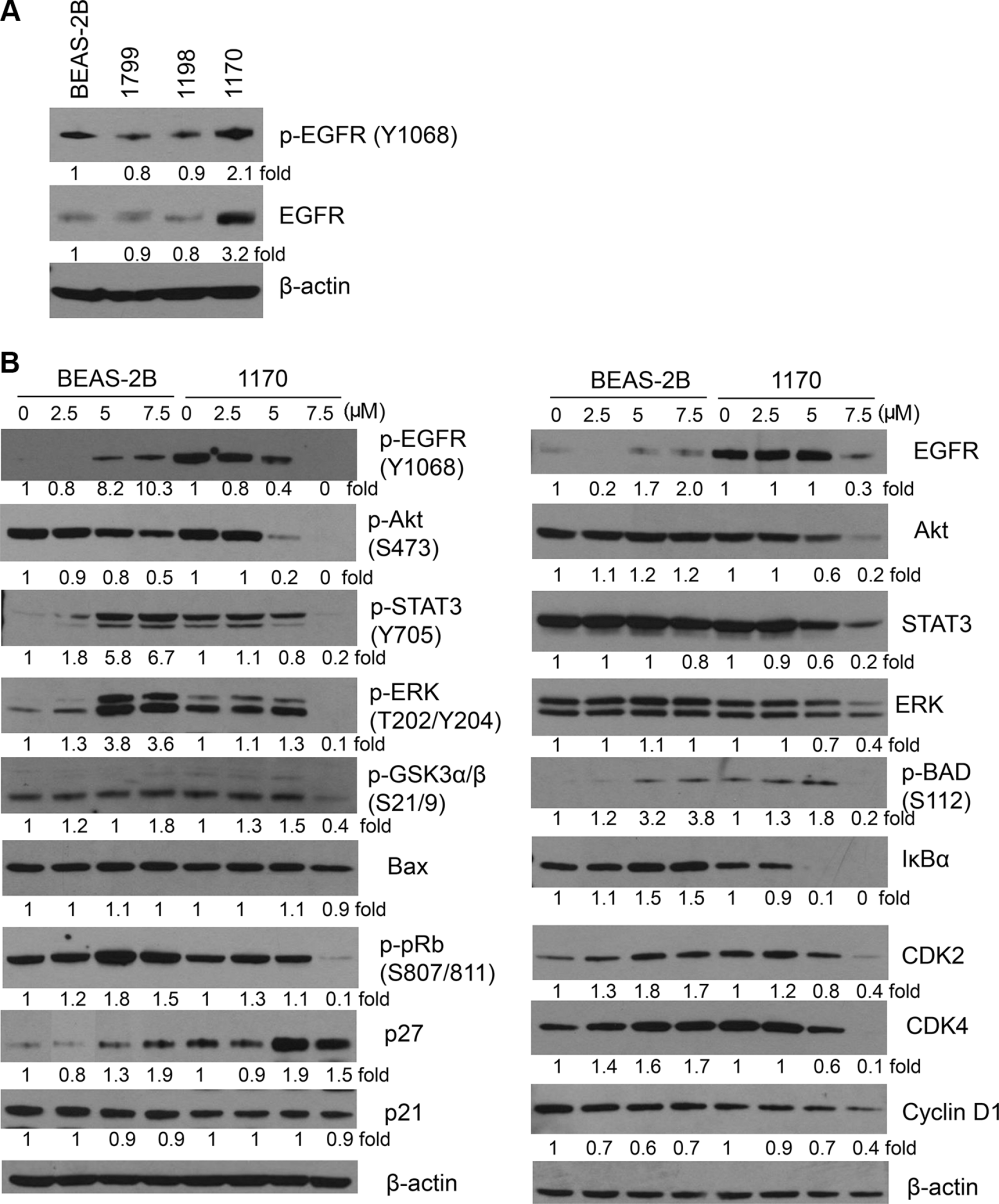
Effect of honokiol on the expression of EGFR and its downstream effector proteins (**A**) Constitutive level of total and phospho-EGFR in parental immortalized BEAS-2B cell line and its premalignant (1799, 1198) and tumorigenic (1170) derivatives. (**B**) Honokiol differentially modulated the level of EGFR and its downstream effectors in 1170 cells in a dose-dependent manner. Cells were treated with the different concentrations of honokiol for 72 h, and cell lysates were analyzed by Western immunoblotting as described in Material and Methods. At least three independent assays were carried out using cell lysates prepared on different days.

To reveal the underlying mechanisms through which honokiol preferentially induced anti-proliferative and proapoptotic effects in 1170 cells, we focused on the EGFR signaling pathway, as our preliminary studies showed a higher constitutive level of total- and phospho-EGFR in these cells compared to the level in BEAS-2B, 1799 and 1198 cells (Figure 2A). In line with the results from MTT and apoptosis assays, exposure of 1170 cells to different concentrations of honokiol (0–7.5 μM) for 72 h induced a dose-dependent reduction in the level of phospho-EGFR, while total EGFR level was reduced only at the highest concentration (Figure 2B). Likewise, honokiol decreased levels of total and phospho- Akt, ERK, and STAT3, and expression of IκBα and cell cycle-related proteins, including cyclin D1, CDK2, CDK4, phospho-pRb, and p27, all of which are downstream effectors of the EGFR signaling pathway. On the other hand, honokiol-treated BEAS-2B cells exhibited an increase in the expression of pro-growth and pro-survival proteins, including phospho-EGFR, phospho-STAT3, phospho-ERK, phospho-pRb, IκBα, CDK2, and CDK4 (Figure 2B).

**Figure 3:**
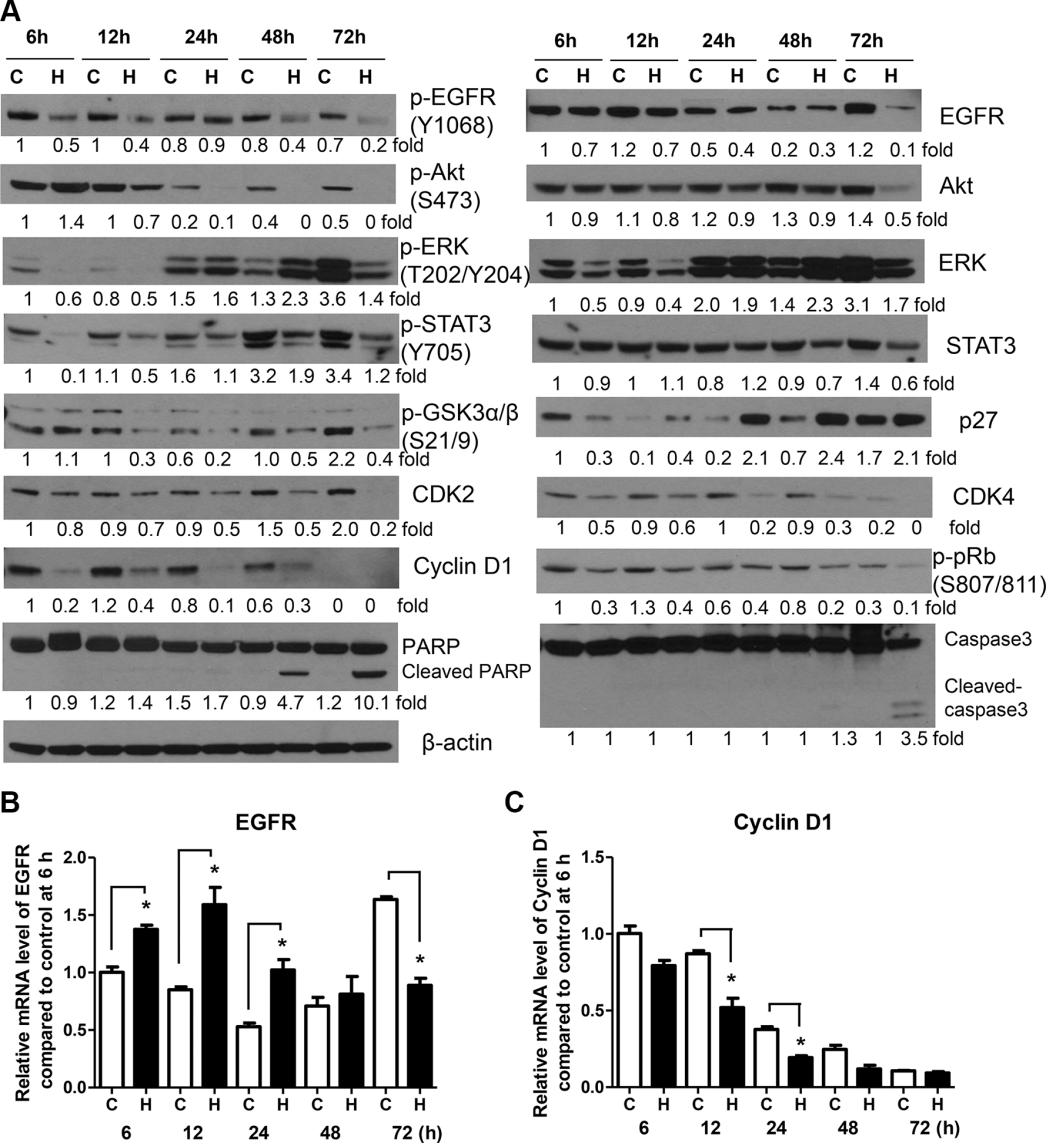
Honokiol modulates the expression of EGFR and its downstream effectors in a time-dependent manner (**A**) Representative Western blots showing time-dependent effects of honokiol on the level of EGFR and its downstream effectors. BEAS-2B and 1170 cells were treated with honokiol (7.5 μM) for 6, 12, 24, 48 and 72 h. Subsequently, cell lysates were prepared and levels of the different proteins determined by Western immunoblotting as described in the Material and Methods section. Analysis of EGFR (**B**) and cyclin D1 (**C**) mRNA levels in 1170 cells treated with honokiol (7.5 μM) for 6, 12, 24, 48 and 72 h. Cells were treated with honokiol, total RNA prepared and the mRNA levels of EGFR and cyclin D1 were measured by quantitative qRT-PCR as described in the Materials and Methods section. For both Western immunoblotting and qRT-PCR assays, at least three independent assays were carried out using cell lysates prepared on different days. C, untreated control; H, honokiol. *P < 0.05, compared with the control group.

To determine honokiol-induced temporal changes in EGFR and its downstream effectors, 1170 cells were treated with the drug for 6, 12, 24, 48 or 72 h and levels of EGFR and its downstream effectors were determined. The expression of phospho-EGFR, phospho-STAT3 and cell cycle-related proteins decreased as early as 6 h after treatment, whereas total EGFR and phospho-Akt were significantly reduced beginning 12 h and 72 h later, respectively (Figure 3A). Total and phospho-ERK exhibited triphasic expression changes in which their levels were decreased during the early time points, followed by recovery 24 h later and then suppression again at 72 h. Cleavage of caspase3 and PARP was observed beginning 48 h after treatment. Overall, the reduction in the expression of phospho-EGFR as early as 6 h suggest that the growth inhibitory and pro-apoptotic effects of honokiol in 1170 cells are mediated via inhibition of EGFR phosphorylation.

To examine whether honokiol-induced suppression of EGFR and cyclin D1 in 1170 cells is mediated via decreased mRNA synthesis, cells were treated with honokiol for different periods of time and EGFR and cyclin D1 mRNA levels were analyzed by qRT-PCR. Compared to the respective controls, cells treated with honokiol exhibited upregulation of EGFR mRNA levels during the early time points (6–24 h), returned to the level in control cells at 48 h and downregulated at 72 h (Figure 3B). On the other hand, honokiol suppressed the mRNA levels of cyclin D1 at all time points, except at 72 h, at which time the expression of the gene reached the lowest level in both control and honokiol-treated cells (Figure 3C). These results are in line with the results from Western immunoblotting studies in that total EGFR and cyclin D1 mRNA levels were decreased at 72 h and as early as 6 h after honokiol treatment, respectively.

### Concentration of EGF in the culture media modulated the anti-proliferative and proapoptotic effects of honokiol and expression/activation of EGFR and its downstream effectors

**Figure 4:**
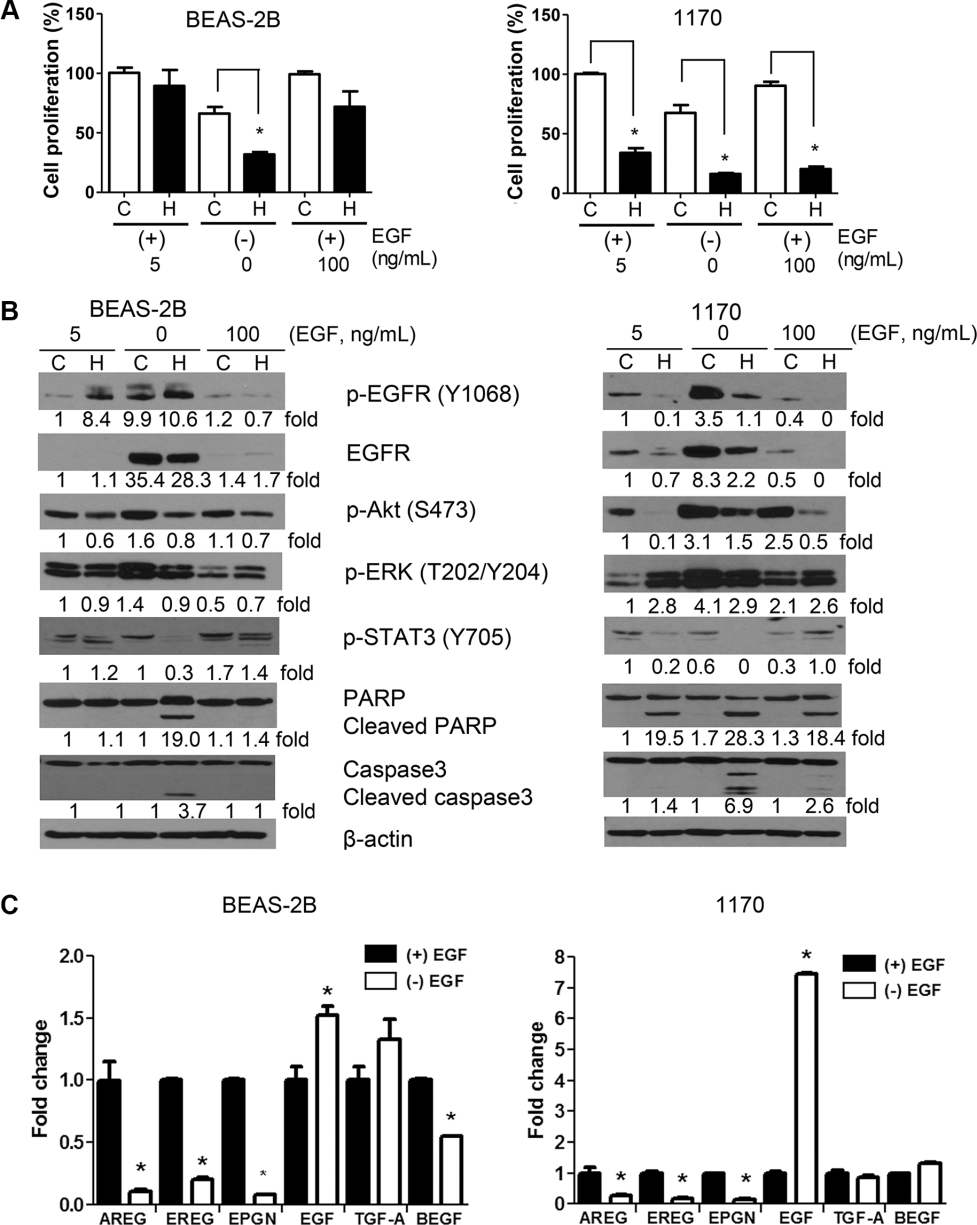
Effect of EGF on the anti-proliferative and pro-apoptotic effects of honokiol (**A**) EGF concentration in the culture media modulated the growth of untreated or honokiol-treated BEAS-2B and 1170 cells. Cells were grown in culture media containing standard amounts of EGF (5 ng/mL), no EGF or excess amounts of EGF (100 ng/mL) with or without honokiol (5 μM) for 48 h and the proliferation of the cells was determined by MTT assay as described in the Materials and Methods section. Data are presented as percentage relative proliferation compared to untreated cells. *P < 0.05, compared with the control group. (**B**) Representative Western blots showing modulation of the level of cell proliferation-and survival-related proteins by the concentration of EGF in the cell culture media. Cells were treated as in Figure 4A and cell lysates were analyzed by Western immunoblotting as described in in the Materials and Methods section. Assays were performed at least three times using cell lysates prepared on different days. (**C**) Expression of EGFR ligands in BEAS-2B and 1170 cells grown in EGF-deficient (−EGF) or EGF-supplemented (+EGF) culture media. Cells were grown for 48 h in culture media in the absence or presence of EGF (5 ng/ml) for 48 h, RNA prepared and the mRNA levels of six EGFR ligands determined by qRT-PCR as described in the Materials and Methods section. Assays were performed at least three times. *P < 0.05, compared with the control group.

The expression of EGFR ligands has been shown to influence treatment response of different cancers, including lung cancer [16–18]. Therefore, we sought to determine if the concentration of EGF in the culture media modulates cell proliferation, the expression of cell proliferation-and survival-related proteins, and the anti-proliferative and pro-apoptotic activities of honokiol in BEAS-2B and 1170 cells. The cell proliferation of BEAS-2B and 1170 cells grown in EGF-deficient culture medium was reduced by about 33% compared to the respective cells grown in culture medium supplemented with the standard concentration of EGF (5 ng/mL, Figure 4A). Honokiol treatment further decreased the proliferation of BEAS-2B and 1170 cells grown in EGF-depleted media and 1170 cells were more sensitive than BEAS-2B cells (75% reduction in the proliferation of 1170 cells versus 52% reduction in BEAS-2B cells, compared to untreated cells). Growing cells in EGF-deficient media also caused a dramatic increase in the expression and activation of EGFR and its downstream effectors Akt and ERK, particularly in 1170 cells, but these effects were remarkably abrogated by honokiol and the cells underwent apoptosis as demonstrated by cleavage of caspase 3 and PARP (Figure 4B).

The proliferation of BEAS-2B and 1170 cells cultured in the presence of excess EGF (100 ng/ml) was similar to that of cells grown in media containing standard amounts of EGF (5 ng/mL, Figure 4A). Despite the 20-fold increase in the concentration of EGF, the expression/activation of EGFR was significantly lower than that observed in cells grown in standard amounts of EGF (Figure 4B). On the other hand, levels of phospho-Akt and phospho-ERK were higher in 1170 cells grown with excess EGF. Upon treatment with honokiol, the proliferation of 1170 cells grown in culture media containing excess EGF was dramatically reduced (70% reduction), whereas only moderate effects were observed in BEAS-2B cells (28% reduction) grown under the same conditions (Figure 4A and 4B). These results are consistent with the differential suppression of phospho-Akt, and an increase in PARP and caspase3 cleavage in 1170 cells, but not in BEAS-2B cells (Figure 4B). Overall, these results indicate that overexpression of EGF does not affect the anti-proliferative and proapoptotic effects of honokiol.

To examine autocrine secretion of EGFR ligands by BEAS-2B and 1170 cells and if this modulates the anti-proliferative and pro-apoptotic effects of honokiol in these cells, we compared the mRNA level of 6 EGFR ligands [amphiregulin (AREG), epiregulin (EREG), epigen (EPGN), EGF, transforming growth factor alpha (TGF-α), and hepatic-binding EGF like growth factor (HBEGF)] in BEAS-2B and 1170 cells grown in a basal media containing standard amounts of EGF (5 ng/mL) or EGF-deficient culture media in the absence or presence of honokiol. As depicted in Figure 4C, whereas mRNA levels of AREG, EREG, and EPGN levels were dramatically reduced in cells cultured in EGF-deficient culture media, the mRNA expression of EGF increased by 1.5- and 7-fold in BEAS-2B and 1170 cells respectively. In BEAS-2B cells, but not in 1170 cells, levels of TGF-α and HBEGF were slightly increased and decreased, respectively. Honokiol did not modulate the expression of the ligands (data not shown).

### Honokiol was more effective than EGFR siRNA in abrogating AKT phosphorylation in 1170 cells

**Figure 5:**
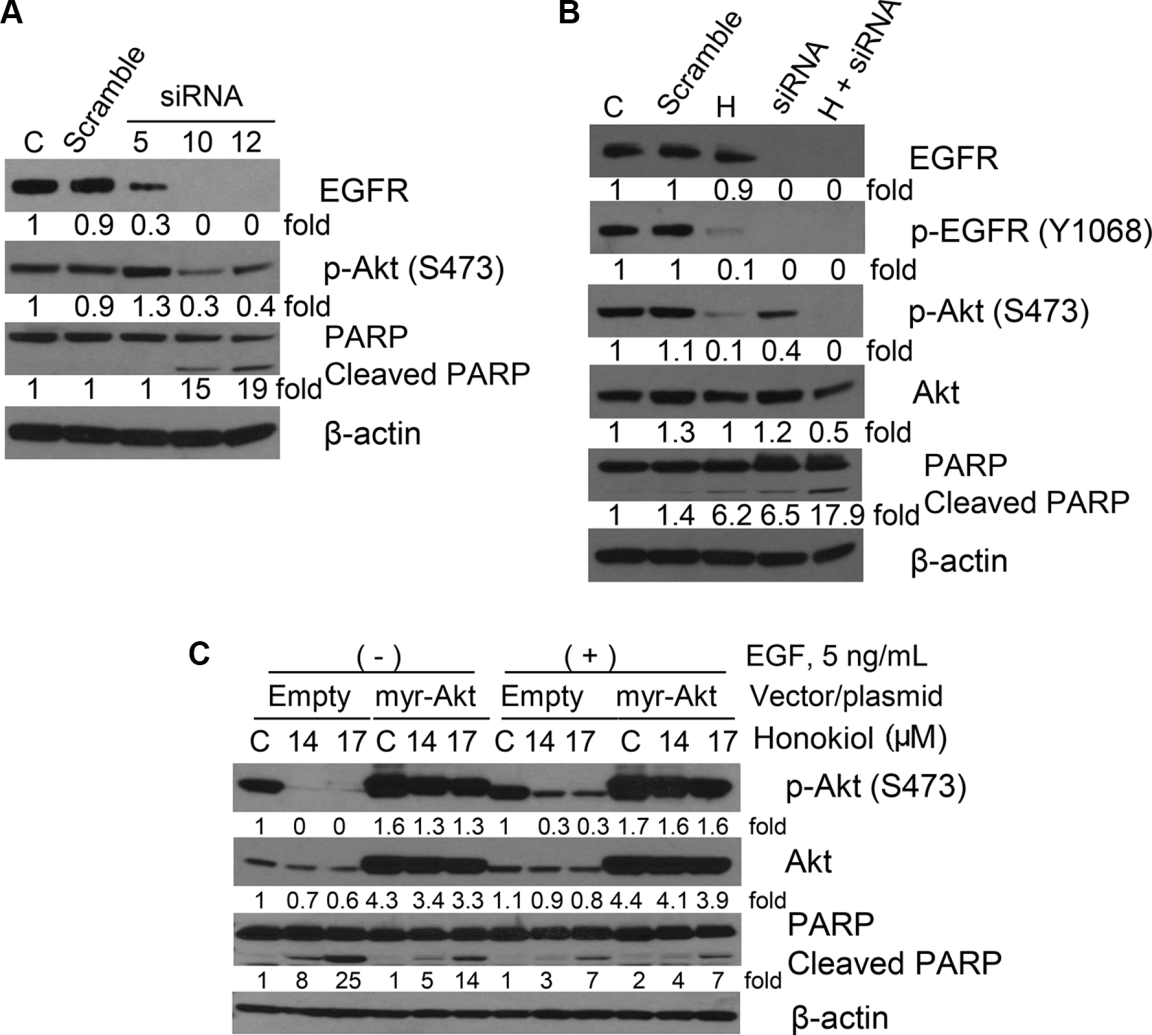
Representative Western immunoblotting results showing effects of EGFR siRNA or AKT overexpression on the expression of cell proliferation- and apoptosis-related proteins in 1170 cells (**A**) Cells were transfected with different EGFR siRNAs for 6 h, media changed to fresh media, and cells incubated until 48 h. (**B**) Cells were transfected with EGFR siRNA 10 as in Figure 5A, media changed to fresh media containing DMSO or honokiol (10 μM) at day 1 and cells further incubated for another day. (**C**) Cells were transfected with empty vector or a vector containing myr-Akt (0.5 μg) for one day and further incubated with DMSO or honokiol (14 and 17 μM) for another day. For all studies, cell lysates were prepared and Western immunoblotting assays performed as described in the Materials and Methods section. C: untreated control; H: honokiol.

To examine the role of EGFR silencing on the expression of its downstream targets and if this would modulate the anti-proliferative and proapoptotic effects of honokiol in 1170 cells, we employed siRNA technology to knock down EGFR. First, we assessed the effect of EGFR siRNA on EGFR expression, Akt activation and PARP cleavage. As depicted in Figure 5A, among the three EGFR siRNAs (Hs_EGFR 5, 10 and 12), two of them (Hs_EGFR 10 and 12) completely abolished EGFR expression, significantly decreased phospho-Akt levels and caused PARP cleavage, indicating that EGFR was silenced. In subsequent studies, 1170 cells were transfected with scramble siRNA or Hs_EGFR 10, and treated with DMSO or honokiol (10 μM) for 24 h and expression and activation of EGFR and Akt and PARP cleavage were determined. As expected, EGFR siRNA completely abrogated the expression of total and phospho-EGFR, but only moderately reduced Akt phosphorylation (Figure 5B). On the other hand, although honokiol did not affect the level of total EGFR and less efficiently reduced EGFR phosphorylation, as compared to EGFR siRNA, Akt phosphorylation was almost completely abrogated, suggesting EGFR-independent suppression of phospho-Akt by honokiol. Cells transfected with EGFR siRNA and treated with honokiol exhibited complete abolition of phospho-Akt and a higher level of PARP cleavage compared to cells with silenced EGFR or cells treated with honokiol alone (Figure 5B).

To further examine the effect of honokiol on Akt, 1170 cells were transfected with an empty vector or myr-Akt and grown in EGF-deficient cell culture media or media supplemented with EGF. As shown in Figure 5C, overexpression of myr-Akt, a constitutively active form of Akt, dramatically increased AKT phosphorylation and attenuated the effects of honokiol on Akt phosphorylation and PARP cleavage. These effects were more marked in cells grown in EGF-deficient culture media.

### Computational simulation studies showed a potential binding mode of honokiol in EGFR

To provide an in-depth picture of the interaction between honokiol and the active domain of EGFR, we employed molecular docking, Molecular Mechanics/Generalized Born Surface Area (MM-GBSA) calculations, and a molecular dynamics (MD) simulation.

**Figure 6:**
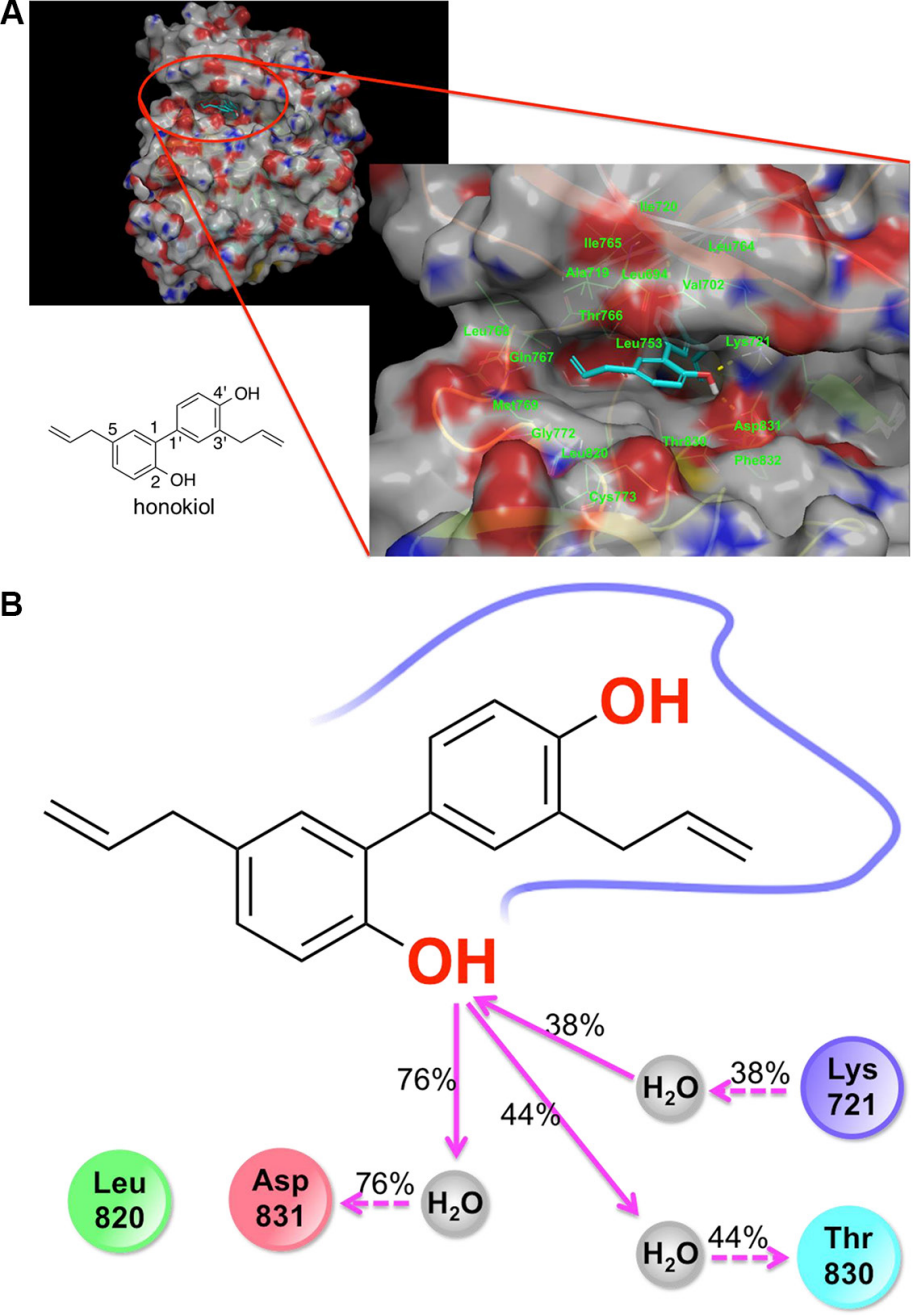
Molecular docking and molecular dynamics simulation results showing the binding of honokiol to the kinase domain of EGFR (**A**) Docking pose of honokiol in the kinase domain of the EGFR. Gray color represents carbon and hydrogen. Blue color indicates nitrogen atom. Red color represents oxygen. (**B**) 2D interaction map of honokiol in the kinase domain of EGFR as obtained from molecular dynamics simulation studies: red, negatively charged amino acid; blue, positively charged amino acid; cyan, polar amino acid; green, hydrophobic amino acid; gray, water; pink solid arrow, H-bond with the backbone of the protein; pink dotted arrow, H-bond with the side chains of the amino acid residues of the protein; % values, the percent of the MD simulation time in which H-bond was formed.

Overlaying honokiol with EGFR kinase domain inhibitors erlotinib and gefitinib, and a computational solvent mapping of EGFR suggest that the kinase domain of EGFR might be the most likely site for the binding of honokiol (Supplementary Figure S1A and S1B) [19]. Computed relative binding free energy (ΔG) for honokiol, erlotinib and gefitinib suggested that honokiol had a relatively lower binding affinity for EGFR than erlotinib, while erlotinib and gefitinib had virtually the same binding affinity for EGFR (Figure 6A and Supplementary Table S1). Honokiol's hydroxyl groups formed H-bonding interactions with Lys721, Asp831, and Leu764, while its biaryl and allyl groups formed hydrophobic interactions with Met769, Leu820, Thr830, Leu694, Val702, Ala719, Lys721, Thr766, Leu753, L764, and L768. Due to the dynamic nature of the EGFR kinase domain, we further investigated honokiol's binding mode in the EGFR kinase domain by performing MD simulation of the EGFR:honokiol complex (Supplementary Figure S2A). MD simulation showed that the 2-OH of honokiol consistently interacted with Lys721, Thr830, and Asp831 through water-mediated H-bonding interactions (Figure 6B and Supplementary Figure S2B), indicating that water-mediated H-bonding interactions might be crucial for honokiol binding to EGFR.

### Comparative and combinatory anti-proliferative and apoptotic effects of erlotinib and honokiol

**Figure 7:**
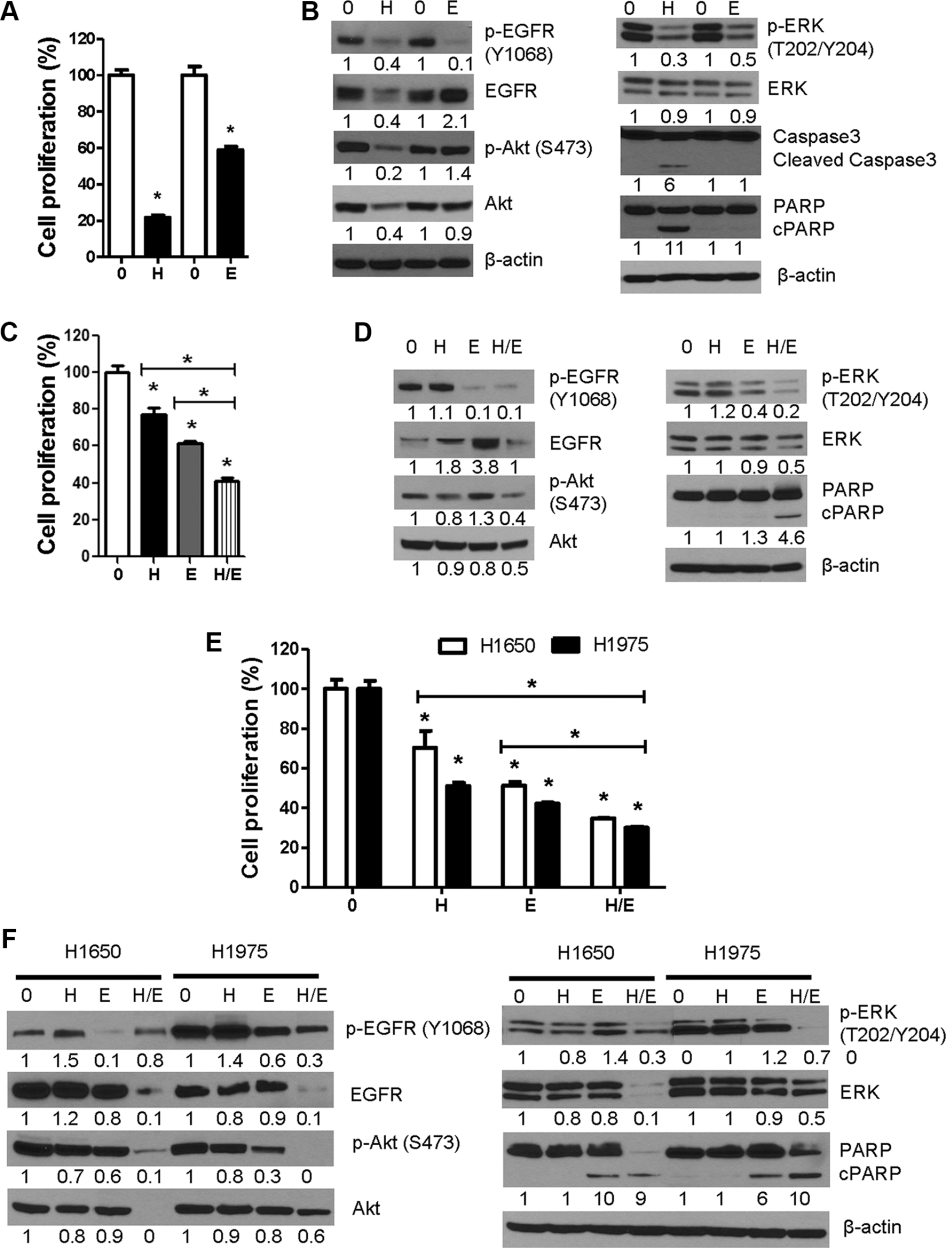
Comparative and combinatory anti-proliferative and pro-apoptotic effects of honokiol and the EGFR TKI erlotinib in 1170 and erlotinib-resistant NSCLC H1650 and H1975 cells (**A**, **B**) Cells were treated with equimolar concentrations of honokiol or erlotinib (7.5 μM) for 48 h and modulation of cell proliferation and expression of cell proliferation-and apoptosis-related proteins were determined by MTT assay (A) or Western immunoblotting (B). (**C**, **D**) Bronchial 1170 cells were treated with honokiol (2.5 μM), erlotinib (5 μM) or honokiol + erlotinib for 48 h and effects on cell proliferation and cell growth- and apoptosis-related proteins were determined by MTT assay (C) or Western immunoblotting (D), respectively. (**E**, **F**), H1965 and H1975 cells were treated with honokiol (10 μM), erlotinib (2.5 μM or 5 μM for H1650 and 1975 cells, respectively) or honokiol + erlotinib for 48 h and modulation of cell proliferation and expression of cell growth-and apoptosis-related proteins determined by MTT assay (E) and Western immunoblotting (F), respectively. Assays were performed at least three times. *P < 0.05, compared with the control group. H: honokiol; E, erlotinib; H/E: honokiol + erlotinib.

To determine if the stronger EGFR binding efficiency of erlotinib, compared to honokiol, is paralleled by higher anti-proliferative and proapoptotic effects, we treated 1170 cells with equimolar concentrations of the two drugs (7.5 μM) for 48 h and the effects on cell proliferation and cell proliferation- and apoptosis-related proteins were determined. As depicted in Figure 7A, unlike the stronger EGFR binding efficiency of erlotinib, its anti-proliferative effects were weaker than that of honokiol (40% and 80% reductions by erlotinib and honokiol, respectively). Similarly, although erlotinib almost completely abrogated phospho-EGFR and moderately reduced phospho-ERK, it did not cause caspase 3 or PARP cleavage. On the other hand, as in the previous assays, honokiol suppressed levels of total- and phospho-EGFR and Akt and phospo-ERK and induced caspase 3 and PARP cleavage (Figure 7B).

To assess if co-treatment with honokiol and erlotinib induces higher anti-proliferative and proapoptotic effects than the individual drugs, 1170 cells and EGFR mutant and erlotinib resistant H1650 and H1975 NSCLC cells were treated with honokiol and erlotinib, individually or in combination, for 48 h. In MTT assay with 1170 cells treated with low concentrations of honokiol (2.5 μM) and/or erlotinib (5 μM), honokiol, erlotinib, and honokiol + erlotinib reduced the proliferation of the cells by 22, 40, and 60%, respectively (Figure 7C). Similarly, in MTT assays with H1650 and H1975 cells, honokiol, erlotinib and honokiol + erlotinib reduced the proliferation of the cells by 30%, 49% and 65% and 49%, 58% and 70%, respectively (Figure 7E). In Western blot studies with 1170 cells, whereas honokiol did not significantly affect the level of any of the proteins, erlotinib markedly suppressed levels of phospho-EGFR and phospho-ERK although these effects did not lead to PARP cleavage. On the other hand, combinatory treatment with the two drugs led to significant reductions in the expression of phospho-EGFR, total-and phospho-Akt, and total-and phospho-ERK and an increase in PARP cleavage (Figure 7D). Likewise, in Western assays with cell lysates with H1650 and H1975 cells, individual treatments with honokiol or erlotinib did not induce significant effects on any of the proteins with the exception of erlotinib-induced modulation of phospho-EGFR, phospho-Akt (H1975 cells) and PARP cleavage. On the other hand, combinatory treatment with honokiol and erlotinib significantly suppressed the expression of total- and phospho-EGFR (H1975 cells), total- and phospho-Akt, total and phospho-ERK and increased PARP cleavage (Figure 7F). Overall, 1170 cells were more sensitive to the anti-proliferative effects of honokiol than H1650 and H1975 cells, whereas the opposite was true for erlotinib. Moreover, combinatory treatment with honokiol and erlotinib induced significantly higher anti-proliferative and proapoptotic activities than honokiol or erlotinib alone in 1170, H1650 and H1975 cells.

### Intranasally administered liposomal honokiol suppressed the multiplicity and growth of NNK-induced lung tumors in A/J mice

**Figure 8:**
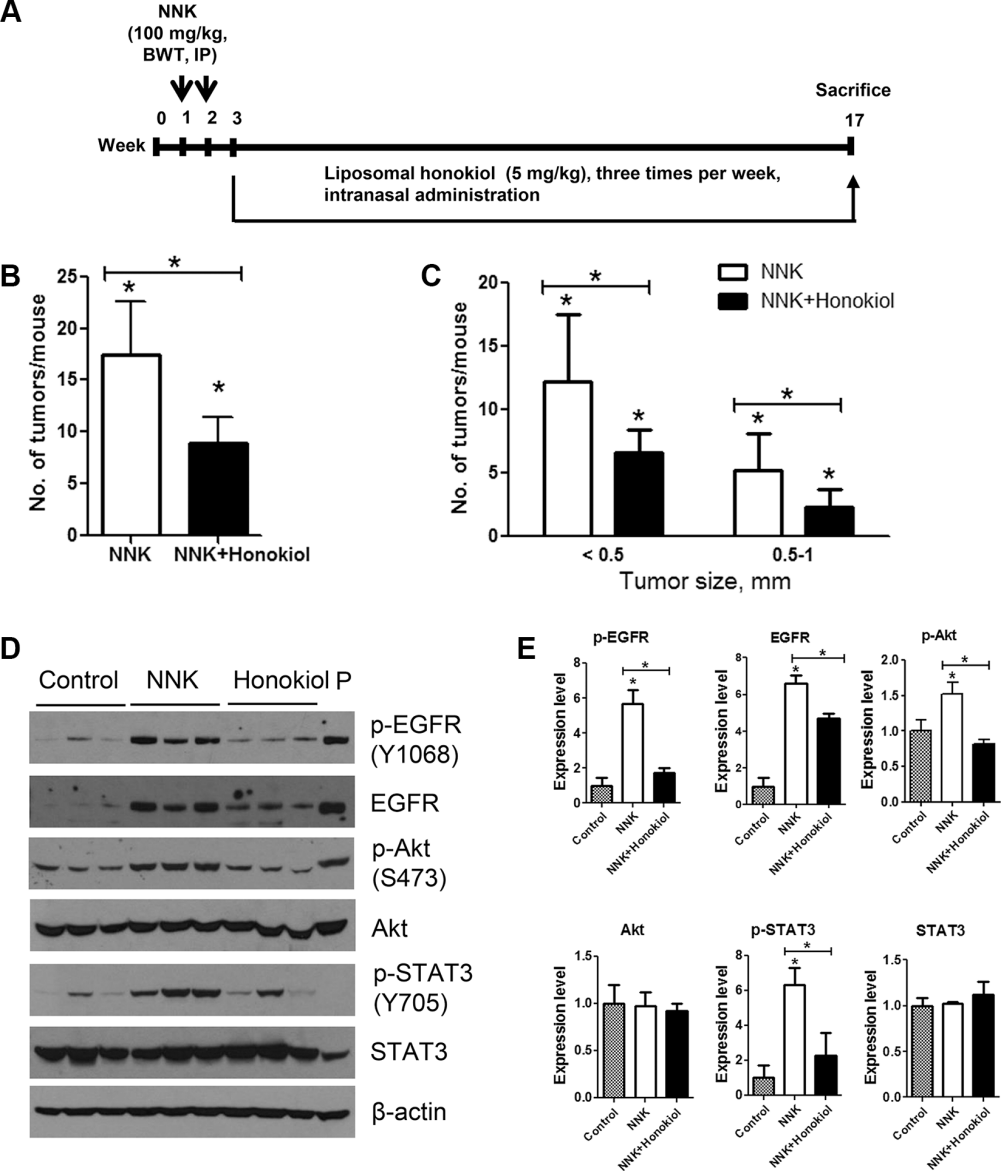
Lung tumor inhibitory effects of intranasally administered liposomal honokiol in A/J mice (**A**) Experimental design of the study. Three groups of female A/J mice (*n* = 15/group) were established. Two groups (NNK control and NNK + honokiol groups) received two doses of NNK (100 mg/kg body weight, ip injection) whereas the vehicle group was injected with physiological saline solution. Beginning 1 week after the final NNK dose, mice in the NNK + honokiol group received liposomal honokiol (5 mg/kg) by intranasal instillation three times a week. Mice in the NNK control group received empty liposome three times a week. The study was terminated at week 17. Upon termination of the study, lungs were harvested, tumors on the surface of the lung were counted (**B**) and the size of the tumors determined (**C**) under stereo microscope. (**D**) Representative western immunoblots showing the effects of honokiol on the expression of EGFR and its downstream effectors. Normal lung tissues (vehicle control group) or lung tumor tissues (from NNK control and NNK + honokiol groups) were randomly selected (three mice/group), tissue lysates prepared and individually analyzed by Western immunoblotting as described in the Materials and Methods section. Immunoblotting studies were repeated three times with different samples. Cell lysates from lung adenocarcinoma A549 cell line were used as positive control and loaded on to the last lane (P). (**E**) Quantification of the western blot results. Densitometry measurements of western blot bands were performed using digitalized scientific software program UN-SCAN-IT software. *P < 0.05, Control group versus NNK group and NNK group versus NNK + honokiol group.

To determine whether the *in vitro* anti-proliferative and proapoptotic effects of honokiol will be paralleled by *in vivo* antitumor effects, mice were pretreated with the lung carcinogen NNK and given intranasal instillations of liposomal honokiol (5 mg/kg), five days a week, for 14 weeks (Figure 8A). Weekly mouse body weight measurements showed that honokiol did not affect weight gain.

Mice treated with NNK and given the vehicle (empty liposome) had an average of 17.4 ± 5.1 lung tumors/mouse, whereas the group treated with NNK and given liposomal honokiol developed 8.9 ± 2.6 lung tumors/mouse, corresponding to a significant reduction of tumor multiplicity by 49% (Figure 8B). Moreover, classification of NNK-induced tumors according to size indicated that liposomal honokiol significantly reduced the growth of both smaller (< 0.5 mm) and bigger lung tumors (0.5–1 mm) (Figure 8C).

We further examined the potential mechanisms through which honokiol suppressed NNK-induced lung tumorigenesis. As shown in Figure 8D and 8E, lung tissues from mice treated with NNK and received empty liposomes had a significantly higher level of total and phospho-EGFR, phospho-Akt and phospho-STAT3 compared to the level in untreated control mice. Intranasal administration of honokiol to NNK-treated mice reduced the expression and activation of EGFR and activation of Akt and STAT3. Levels of total Akt and STAT3 were modulated neither by NNK nor NNK + honokiol. These results are in line with our findings in cell line models.

## DISCUSSION

In this study, we showed that exposure of a unique set of immortalized (BEAS-2B), premalignant (1799 and 1198) and tumorigenic (1170) human bronchial epithelial cell lines with different levels of EGFR (1170 >> 1799 > 1198 = BEAS-2B) [20] to honokiol induced differential anti-proliferative and pro-apoptotic effects in 1170 cells, which express the highest level of EGFR. Although honokiol reduced the proliferation and survival of EGFR-mutant H1965 and H1975 cell lines, its efficacy was weaker than that observed in 1170 cells, whereas the reverse was true for erlotinib. However, simultaneous treatment of 1170, H1650 or H1975 cells with honokiol and erlotinib induced significant combinatory inhibitory effects on cell proliferation and cell proliferation-and survival-related proteins. In line with the *in vitro* effects, intranasal administration of liposomal honokiol to NNK-pretreated mice significantly reduced the multiplicity and growth of NNK-induced mouse lung tumors as well as levels of total- and phospho-EGFR, phospho-STAT3 and phospho-Akt in lung tumors.

EGFR overexpression and mutation have been demonstrated in pre-malignant lung epithelium as well as normal bronchial epithelium adjacent to adenocarcinoma [13–15]. Therefore, targeting EGFR during the early stage of lung tumorigenesis could suppress the development of lung cancer. Currently two main anti-EGFR strategies are in clinical use: low-molecular-weight tyrosine kinase inhibitors (TKIs) that compete with ATP for binding to the tyrosine kinase domain of the receptor, and monoclonal antibodies that are directed at the ligand-binding extracellular domain. Several groups have examined the lung cancer chemopreventive efficacies of EGFR TKIs gefitinib and erlotinib in different preclinical models [21–27]. However, the effects were weak and inconsistent. Moreover, EGFR TKIs are not suitable to be used as chemopreventive agents as they cause severe adverse effects [28]. In a recent study in which erlotinib was used as adjuvant chemopreventive agent in high-risk head and neck cancer patients, treatment was discontinued due to drug-related toxicities [29]. Therefore, it is prudent to develop novel, effective and safe agents for EGFR-targeted chemoprevention. One such promising compound is honokiol, which has been shown to inhibit EGFR in various cancer models and potentiated the anticancer effects of EGFR tyrosine kinase inhibitors without any side effects [5, 30–32].

In the present study, we observed that honokiol-treated BEAS-2B cells exhibited increased expression of phospho-EGFR, phospho-STAT3, phospho-ERK and cell cycle-related proteins, whereas 1170 cells exposed to honokiol showed a significant reduction in the level of all these proteins. Enhanced expression of phospho-EGFR and associated proteins in normal BEAS-2B cells by honokiol is consistent with previous reports which showed that the EGFR pathway coordinates the process of regeneration and recovery of normal lung cells exposed to stress factors [33–36]. Such signal fades away after normal organogenesis and tissue injury/repair to maintain homeostasis [37]. Unlike honokiol, EGFR tyrosine kinase inhibitors erlotinib and gefitinib impair the regeneration of normal pulmonary epithelial cells [38–41] through the blockade of EGFR-dependent phosphorylation, and therefore this group of drugs is commonly incriminated as etiologies of interstitial lung disease, a lung-specific toxicity arising in NSCLC patients receiving these drugs.

Since our results showed that honokiol significantly abrogated EGFR and its downstream effectors in 1170 cells, it is imperative to assess the mechanisms involved. Studies on the time-dependent effects of honokiol on mRNA and protein levels of EGFR showed that although the drug induced significant anti-proliferative effects as early as 24 h after treatment, the mRNA and protein expression of EGFR was not altered until 72 h. On the other hand, levels of phospho-EGFR as well as its downstream effectors phospho-Akt, phospho-STAT3 and cell cycle-related proteins were significantly reduced within 6–12 h. These observations indicate that the anti-proliferative and apoptotic effects of honokiol in 1170 cells are most likely mediated through inhibition of EGFR phosphorylation. Moreover, the results from molecular docking and molecular dynamics simulation studies show that honokiol binds to the kinase domain of EGFR, although the binding affinity of honokiol was less strong compared to that of erlotinib or gefitinib. Despite its weaker EGFR binding affinity, compared to erlotinib, the anti-proliferative activities of honokiol were stronger than that of erlotinib. This could be ascribed to, among other factors, the existence of EGFR-independent inhibitory effects of honokiol. In agreement with this, studies on comparative suppression of Akt phosphorylation by honokiol and EGFR siRNA showed 90% and 60% reductions respectively (Figure 5B). Moreover, previous molecular docking studies indicated that honokiol binds to Akt with an efficiency similar to that of the pan-Akt inhibitor GSK690693 [32]. Honokiol has been shown to block Akt activation resulting from PTEN loss, activating mutations in PIK3CA and K-Ras or amplification of ERB2 [42].

One mechanism through which EGFR family members are deregulated in lung cancer is altered ligand expression with possible formation of autocrine loops [43]. Therefore, targeting EGFR ligands has been proposed to be an important complement to existing anti-EGFR approaches [44]. In this study, we sought to determine the effects of EGF depletion or excess EGF on the proliferation and survival of BEAS-2B and 1170 cells and if the level of EGF in the culture media modulates the anti-proliferative and pro-apoptotic effects of honokiol in these cells. Compared to BEAS-2B and 1170 cells seeded in culture media supplemented with the standard concentration of EGF (5 ng/mL), the growth of cells cultured in EGF-depleted media was significantly reduced (Figure 4A). However, cells grown in EGF-depleted media exhibited an increase in the level of EGFR, phospho-EGFR, phospho-Akt, phospho-ERK, especially in 1170 cells (Figure 4B). Enhanced activation of the EGFR pathway was paralleled by a significant increase in the expression of EGF (1.5- and 7-fold increase in BEAS-2B and 1170 cells, respectively), compared to cells grown in EGF-supplemented culture media (Figure 4C), suggesting the presence of an autocrine loop in which EGFR activates the expression of EGF ligand, which in turn activates EGFR. Indeed, bronchial cells have been shown to synthesize several EGF ligands which subsequently are shed by metalloproteinase-dependent cleavage and act as biologically active ligands [24, 45–47]. BEAS-2B and 1170 cells grown in EGF-deficient media were clearly more sensitized to the effects of honokiol as demonstrated by a marked decrease in cell proliferation, suppression of EGFR, phospho-EGFR, phospho-Akt, phospho-ERK and phospho-STAT3 and an increase in the cleavage of caspase-3, −8 and −9 as well as PARP, compared to cells grown in the same culture media and treated with DMSO (Figure 4B).

Contrary to our expectation, 1170 cells grown in culture media containing excess amounts of EGF (100 ng/mL) exhibited reduced levels of total- and phospho-EGFR, compared to cells grown cells grown in EGF-deficient media or media supplemented with the standard amount of EGF. These findings are in line with the reports in human mammary epithelial cells in which cells treated with EGF (100 ng/ml) exhibited faster EGFR degradation via a negative feedback loop in which EGF stimulation phosphorylated Akt and the latter activated mammalian type III PtdInsP kinase that facilitated EGFR degradation [48]. Increased phosphorylation of Akt and ERK in cells grown in the presence of excess EGF could indicate that internalized EGFR can signal from endosomes as described previously [49].

Increased Akt activation is one mechanism through which lung cancer cells develop resistance towards EGFR TKIs [50, 51]. Therefore, combinatory treatment with EGFR TKIs and inhibitors of Akt could be a promising therapeutic strategy to overcome EGFR resistance. In this study, we first observed that the anti-proliferative and pro-apoptotic activities of honokiol in 1170 cells were much stronger than those induced by erlotinib and co-treatment of 1170 cells with honokiol and erlotinib caused combinatory anti-proliferative and pro-apoptotic effects. Subsequently, these studies were extended to erlotinib-resistant H1650 and H1975. Simultaneous treatment of H1650 and H1975 cells with honokiol and erlotinib caused significantly higher growth inhibitory activities and more potent modulatory effects on phospho-EGFR (H1975 cells), total EGFR, total and phopho-Akt, total and phospho-ERK and PARP cleavage, compared to treatment with honokiol or erlotinib alone, indicating the promise of combinatory treatment with erlotinib and honokiol to improve the clinical response of patients with erlotinib-resistant lung cancer. In earlier studies using HNSCC models, honokiol has been shown to enhance the anti-proliferative and anti-invasion activities of erlotinib and tumor growth inhibitory activities of cetuximab [31].

In agreement with the results in cell line models, honokiol significantly suppressed lung tumor burden and activation of EGFR, Akt and STAT3 in NNK-induced lung tumor model bearing *KRAS* mutation [52–54], suggesting that honokiol could target EGFR in NSCLC patients with a history of tobacco smoke and *KRAS* mutation. Many studies in human lung adenocarcinoma patients showed that *KRAS* and *EGFR* mutations are mutually exclusive and *KRAS* confers resistance to treatment with EGFR TKIs [55, 56]. On the other hand, studies with Kras^LA1^ mice, which develop lung adenocarcinoma through somatic activation of a *KRAS* allele carrying an activating mutation in codon 12 suggested that the presence of *KRAS* mutations was not sufficient to confer resistance to EGFR inhibition [24]. These discrepancies could be related to the stage of lung tumorigenesis (targeting of early stage lung tumorigenesis in mouse models versus late stage disease in human patients).

One limiting factor for the further development of honokiol as anticancer drug is its poor bioavailability due to poor gastrointestinal absorption, biotransformation in the liver, secretion in the bile, extensive protein binding and fast excretion [57]. However, this problem has been overcome by developing PEGylated liposomal formulations of honokiol, which increased not only the bioavailability of the drug but also its solubility and half-life [7, 8]. The dose of liposomal honokiol used in this study was determined on the basis of a previous study [8] in which administration of liposomal honokiol to mice at a dose of 25 mg/kg body weight resulted in a plasma concentration of 10 μg/ml. Since our previous studies with diindolylmethane and dimethyl aminoparthenolide indicated that intranasal delivery of drugs improves pulmonary bioavailability [58, 59], and hence we reduced the dose of liposomal honokiol by five-fold to 5 mg/kg. At this dose level, the pulmonary level of honokiol is estimated to be in the range of honokiol concentrations used for the *in vitro* assays (5–7.5 μM).

In summary, this study showed differential anti-proliferative and proapoptotic effects of honokiol in EGFR overexpressing cells and similar effects were observed in NNK mouse model of lung tumorigenesis in which total and phospho-EGFR, phospho-Akt and phospho-STAT3, downstream effectors of EGFR, were suppressed. Moreover, honokiol and erlotinib exhibited combinatory anti-cancer effects in 1170 cells and EGFR mutant cell lines H1650 and H1975 as demonstrated by significant abrogation of phospho-Akt and phospho-ERK expression and an increase in PARP cleavage. Given that EGFR is overexpressed or mutated in most NSCLC cases, leading to an increased EGFR phosphorylation, and that acquired resistance to EGFR TKIs is commonly observed in NSCLC patients who initially respond to these drugs, honokiol is a promising agent for the prevention and therapy of pulmonary malignancies associated with EGFR deregulation alone or in combination with EGFR TKIs.

## MATERIALS AND METHODS

### Chemicals and reagents

Honokiol (≥ 98% of purity) was purchased from LKT laboratories (Minneapolis, MN). Anti-phospho-EGFR (Y1068), anti-total EGFR, anti-phospho-Akt (S473), anti-total Akt, anti-phospho-ERK (T202/Y204), anti-total ERK, anti-phospho-STAT3 (Y705), anti-total STAT3, anti-cyclin D1, anti-CDK2, anti-CDK4, anti- phospho-Rb (S807/811), anti-p27, anti-caspase 3, anti-phospho-GSK3α/β (S21/9), anti-IκBα, anti-bax, anti-phospho-Bad, anti-β-actin, anti-caspase 8, anti-caspase 9 and goat anti-rabbit IgG secondary antibody were from Cell Signaling Technology (Beverly, MA). Anti-poly (ADP-ribose) polymerase (PARP) and anti-p21 were obtained from Santa Cruz Biotechnology. Erlotinib (99% of purity) was purchased from LC Laboratories (Woburn, MA). NNK (99% of purity) was synthesized as described elsewhere [60].

### Cells and cell culture

Immortalized bronchial epithelial cell line 2B (BEAS-2B) and its premalignant (1799 and 1198) and malignant (1170) derivatives were provided by Dr. Klein-Szanto (Fox Chaser Cancer Center, Philadelphia). Cell line 1799 was developed from BEAS-2B cells explanted along with beeswax pellets into rat tracheas that had been denuded of bronchial epithelium and further transplanted into the dorsal subcutaneous tissues of nude mice [20]. Cell lines 1198 and 1170 were developed in a similar manner except that the beeswax pellets contained cigarette smoke condensate. All four cell lines have been tested for mycoplasma infection and were authenticated by short tandem repeat method at MD Anderson's Cell Line Core Facility in Feb., 2015. The EGFR mutant H1650 and H1975 cells were kindly provided by Dr. Shujun Liu, Hormel Institute, University of Minnesota, in 2015. H1650 cells have an in-frame deletion in the EGFR tyrosine kinase domain (EGFR tyrosine kinase domainΔE746-A750, exon 19). H1650 cells have also a deletion of the 3′ part of exon 8 and the entire exon 9 of PTEN, which causes loss of the protein. The cell line H1975 has a sensitizing L858R kinase domain mutation in exon 21, but also a second mutation (T790M, in cis, in the kinase domain) rendering them resistant to the reversible TKIs gefitinib and erlotinib. Bronchial cells were maintained in keratinocyte serum-free medium with recommended supplements (Life Technologies Inc., Gaithersburg, MD) in 5% CO_2_ incubator, whereas H1650 and H1975 cells were grown in RPMI supplemented with 2.5–10% of serum and 1% penicillin-streptomycin under the same condition.

### Cell viability assay

The effect of honokiol on the cell viability was determined by methylthiazole tetrazolium (MTT; Biotium, Hayward, CA) assay as described previously [61].

### Annexin V/Propidium iodide apoptosis assay

To determine the apoptotic effects of honokiol in BEAS-2B and 1170 cell lines, each cell line was treated with different concentrations of the drug (0–7.5 μM) for 72 h. Subsequently, 1 × 10^6^ cells were washed twice with cold phosphate-buffered saline and stained with 5 μL Annexin V-fluorescein isothiocyanate and 5 μL PI (BD Pharmingen, San Diego, CA) for 15 min at room temperature in the dark. The proportion of apoptotic cells was determined by BD LSRII flow cytometer.

### Quantitative reverse transcription–PCR analysis of EGFR, cyclin D1, and EGFR ligands (AREG, EREG, EPGN, EGF, TGF-α, and BEGF)

Total RNA was extracted from BEAS-2B or 1170 cells using the miRNeasy Mini Kit (Qiagen, Valencia, CA) according to the manufacturer's instruction. Quantitative reverse transcription–PCR (qRT-PCR) was performed by Applied Biosystems 7500 fast real-time PCR system as described previously [61] using the gene-specific primers shown in Supplementary Table S2. Comparative Ct method was used to assess the relative gene expression. Values were expressed as relative mRNA expression in honokiol-treated cells compared to DMSO-treated cells.

### Small interfering RNA (siRNA)-mediated knockdown of EGFR

1170 cells were seeded in 60 mm dish at 30% confluency. After overnight culture, cells were transfected with 50 nM of Hs_EGFR_5, 10 and 12 FlexiTube siRNA (Qiagen) and lipofectamin RNAiMAX reagent (Invitrogen) according to manufacturer's instructions. Forty-eight hours after transfection, cells were harvested, and EGFR, phospho-Akt and PARP cleavage levels were determined by Western immunoblotting. For the assay in which cells were treated with EGFR siRNA and honokiol, cells were transfected with scrambled siRNA or EGFR siRNA, incubated for 24 h with or without honokiol (10 μM) and then expression and activation of EGFR and Akt and PARP cleavage were determined by Western immunoblotting.

### Western blot analysis of cell lines and mouse lung tissues

For the preparation of cell lysates, DMSO-, honokiol and/or erlotinib-treated cells were incubated in 1 × RIPA buffer with protease- and phosphatase-inhibitor (Pierce, Rockford, IL) for 10 min on ice. Mouse lung tissue lysates were prepared by homogenizing normal lung tissues or microdissected tumors, from three mice in each group, in 1 × RIPA buffer containing protease- and phosphatase-inhibitor. Subsequently, cell and tissue lysates were centrifuged at 14,000 g for 10 min at 4°C, and the supernatants collected and stored at −80°C. Western immunoblotting was performed using protein samples from cell or tissue lysates as described previously [61]. For quantitative determination of protein levels, densitometric measurements of Western blot bands were performed using UN-SCAN-IT software (Silk Scientific, Orem, Utah).

### Preparation of liposomal honokiol

PEGlyated liposomal honokiol was prepared following the method described previously [8] with slight modifications. Briefly, the mixture of phosphatidyl choline, cholesterol, 1,2-distearoyl-sn-glycero-3-phosphoethanolamine-N-[methoxy(polyethylene glycol) −2000] and honokiol in weight ratios of 1:0.15:0.24:0.22 were dissolved in 6 mL chloroform/methanol at a ratio of 3:1 (v/v). The mixture was gently warmed to 40°C, and the solvent evaporated under vacuum until a thin lipid film was formed. The dried lipid films were sonicated in 5% (w/v) glucose solution and lyophilized. Blank liposomes were synthesized by the same method without the addition of honokiol. The diameter of the liposomes was measured by Delsa™ Nano C (Beckman Coulter Inc., Fullerton, CA, USA) and found to be ~105 nm (polydispersity index-0.26). For further studies, liposomal honokiol and blank liposome were dispersed in 5% glucose solution.

### Tumor bioassay

Female A/J mice (6 weeks old) were obtained from The Jackson Laboratory (Bar Harbor, ME) and housed with wood chip bedding in environmentally controlled, specific-pathogen-free animal quarters with a 12-hour light–dark cycle and a relative humidity of 50%. Drinking water and diet were supplied *ad libitum*. The study was approved by the Institutional Animal Care and Use Committee of the University of Minnesota. One week after arrival, the mice were randomized into three groups (10 mice for the vehicle control group and 15 mice each for the NNK and NNK + honokiol groups) and treated with NNK (two doses of 100 mg/kg, once a week, in 0.3 mL physiological saline solution) or the vehicle by intraperitoneal injection. Mice in the NNK + honokiol group received liposomal honokiol (5 mg/kg, in 50 μL physiological saline solution) by intranasal instillation three times a week, from one week after the last dose of NNK until the termination of the study at week 16. This dose level of honokiol is more than 50-fold lower than the dose of the liposomal honokiol agent administered by intraperitoneal injection to mice in earlier studies [8]. Control (negative control) and NNK (positive control) treated mice were treated with the blank liposome in a similar manner. Body weights were determined every week. At the end of the study, the mice were euthanized by carbon dioxide asphyxiation. Subsequently, the lungs were harvested and tumors on the surface of the lung counted and their sizes determined under a dissecting microscope. Lung tissues used for Western blot studies were stored at −80°C. All animal experiments were performed according to the U.S. National Institutes of Health (NIH) Guide for the Care and Use of Laboratory Animals, and approved by the Institutional Animal Care and Use Committee, the University of Minnesota.

### Molecular docking and molecular dynamics simulations to study the binding mode of honokiol to EGFR

The crystal structure of EGFR and erlotinib (PDB ID code 4HJO) was imported to the Maestro workspace and prepared using the protein preparation wizard in Schrodinger Suite [62]. Following the protein preparation process, a grid was generated by selecting erlotinib as a grid center. Then, docking simulations of honokiol were performed in the SP and XP modes in Glide [63]. For molecular dynamics simulation, the complex of the EGFR with the best docking pose of honokiol was taken for the generation of the system files. The simulation box was generated with the dimension of 10Å × 10Å × 10Å and 0.15 M MgCl_2_ was included. The initial short time scale MD simulation (1.2 ps) was performed using desmond [64]. Following this, 20 ns MD simulation of the EGFR complex with honokiol was performed by employing 128 cores. Relative binding free energies of the compounds were calculated using molecular mechanics-generalized Born surface area (MM-GBSA) method (Prime, version 4.1, Schrodinger, LLC, New York, NY). MM-GBSA calculation tool in Schrodinger Suite was used with the VSGB solvation model and OPLS2005 force field. Protein flexibility was defined by calculating flexible residue distances (5Å) from the ligands.

### Statistical analysis

Data for tumor bioassay, western blot analysis and MTT assay are reported as mean ± SD of triplicate determinations. Between-group comparisons were performed using one-way ANOVA and two-tailed *t-test* in Graphpad Prism 5 software (Graphpad, La Jolla, CA). *P*-values < 0.05 were considered significant.

## SUPPLEMENTARY MATERIALS TABLES AND FIGURES


