# Supplementary material for: Honokiol suppresses lung tumorigenesis by targeting EGFR and its downstream effectors
Source: Oncotarget. 2016 Jul 21;7(36):57752–69. doi: 10.18632/oncotarget.10759 (PMC5295387; doi:10.18632/oncotarget.10759)
Supplement: Supplementary file 1 [file oncotarget-07-57752-s001.pdf]

Honokiol suppresses lung tumorigenesis by targeting EGFR and its downstream effectors

Supplementary Materials

Supplementary Table S1: Computed relative binding free energies of erlotinib, gefitinib, and honokiol for EGFR

Compound	Relative binding free energy (ΔG, kcal/mol)			Kd (nmol/L)*
	XP gscore	Glide emodel	MM-GBSA	
Erlotinib	−9.8	−95.0	−91.1	0.97
Gefitinib	−10.5	−94.9	−94.9	0.94
Honokiol	−7.4	−59.0	−79.7	-

*Kd for EGFR kinase domain was obtained from binding DB (www.bindingDB.org).

Supplementary Table S2: Primer information used for qRT-PCR

Gene name	Forward primer (5′-3′)	Reverse primer (5′-3′)
EGFR	GCTGGATGATAGACGCAGATAG	GAAGTTGGAGTCAGGACTTG
CyclinD1	CCTGGTGAACAAGCTCAAGT	GTGTTTGCGGATGATCTGTTTG
β-actin	ACGGTCAGGTCATCACTATC	ACTGTGTTGGCATAGAGGTC
AREG	CTCGGGAGCCGACTATGA	TGCATGTTACTGCTTCCAGG
TGF-α	TCGCTCTGGGTATTGTGTT	CAACGTACCCAGAATGGC
EGF	TGGGCAAGACCTCCAG	GATGACATCGTTTCCCATCAG
EREG	TCCACGTGTGGCTCAAG	TCACGGTCAAAGCCACATAC
BEGF	TGTATCCACGGACCAGC	TCCGAAGACATGGGTCC
EPGN	CAACGCAATGACAGCACTG	TCTAGCTCATGGTGGAATGC

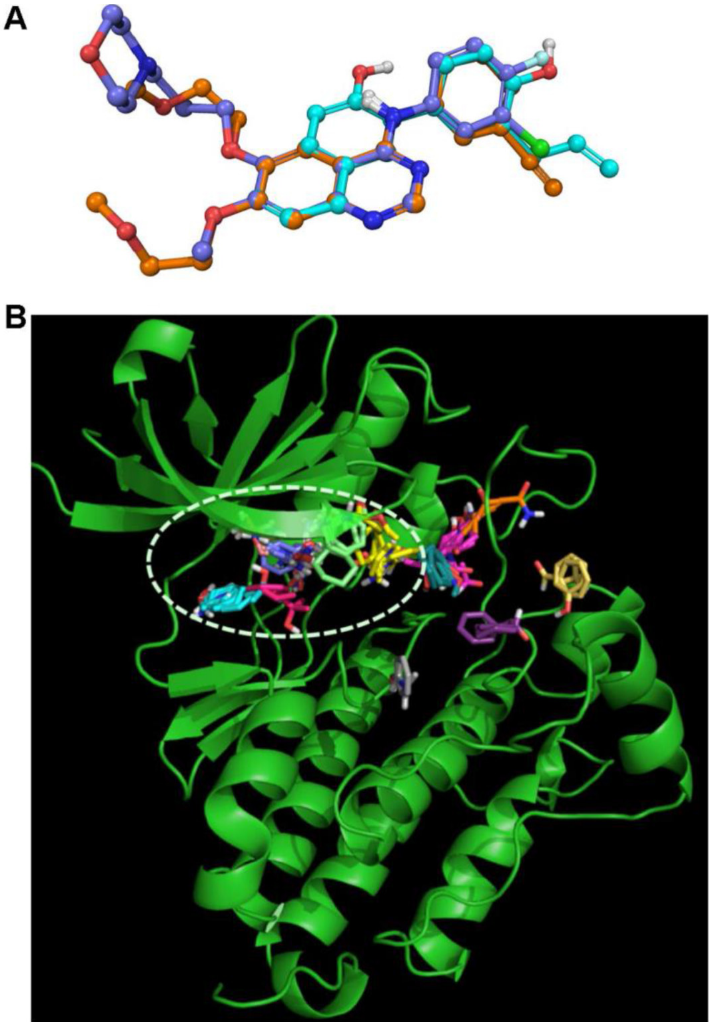

Supplementary Figure S1: Determination of the site for docking simulations. (**A**) Overlay of honokiol (cyan carbon), gefitinib (blue carbon) and erlotinib (orange carbon). (**B**) FTmap of EGFR: most druggable site is displayed with the circle. These analyses were performed in the FTMap server (ftmap.bu.edu).

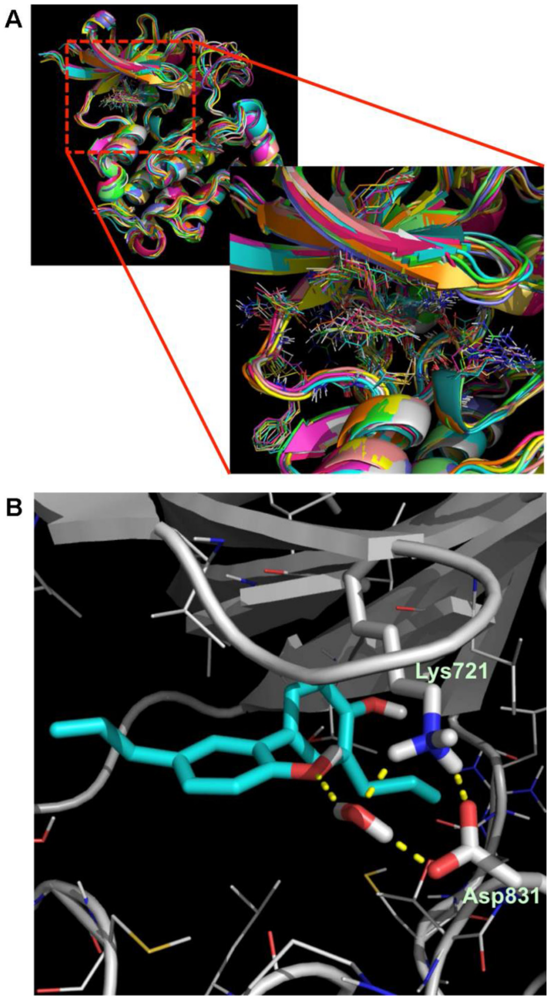

Supplementary Figure S2: Predicted binding poses of honokiol obtained from the MD simulation. (**A**) Snapshots from the MD simulation of the complex of EGFR:honokiol. (**B**) Water-mediated H-bonding network among honokiol, Lys721 and Asp831.
